# Case Report: Winkelmann hip rotationplasty as a last-resort solution

**DOI:** 10.3389/fsurg.2024.1433291

**Published:** 2025-01-07

**Authors:** Robin Evrard, Othmane Miri, Valérie Lacroix, Pierre-Louis Docquier, Thomas Schubert

**Affiliations:** ^1^ Service de Chirurgie Orthopédique et Traumatologique, Cliniques Universitaires Saint-Luc, Bruxelles, Belgique; ^2^ Service de Chirurgie Vasculaire et Thoracique, Cliniques Universitaires Saint-Luc, Bruxelles, Belgique

**Keywords:** hip rotationplasty, limb salvage, surgery, orthopedic oncology, case series

## Abstract

**Background:**

Rotationplasty, an invasive surgery, serves as an alternative to amputation in pediatric orthopedic oncology. It is currently applied in broader cases (e.g., infection, trauma, or malignant tumors). Winkelmann Type BII rotationplasty is a rare procedure with limited literature. Furthermore, no description of rotationplasties where the femur is attached to the sacroiliac joint has been published to date.

**Methods:**

Between September 2022 and March 2023, three patients underwent Type BII rotationplasty. We used the Clavien-Dindo classification to describe postoperative complications and the musculoskeletal tumor society score (MSTS) for functional result assessments.

**Results:**

One patient suffered from multiple complications during the first 6 months postoperatively, one presented a single complication, and one had no complications after 4 and 3 months postoperatively, respectively. Two patients could walk pain-free with the help of crutches. One patient developed a crack on the femur, which did not require surgical revision. They all achieved satisfactory joint amplitudes of at least 50° in passive hip flexion. Unfortunately, one of the patients suffered from lung metastases.

**Conclusions:**

Winkelmann's Type BII rotationplasty is a reliable alternative to hindquarter amputation. Furthermore, we demonstrated that complete resection of the iliac wing and femur fixation through the sacroiliac joint is feasible.

## 1 Introduction

Rotationplasty is a surgical procedure that involves rotating a segment of the limb by 180° and reattaching it in a new position to create a functional joint. Initially developed for severe congenital deformities, this concept has been refined over time. The earliest documented cases were described by Borggreve in 1930 (1). Then, refinements were done by Van Nes and Winkelmann (2–5). While rotationplasty remains a form of sub-amputation, it can lead to good functional outcomes (6–13). The best-known rotationplasty, described by Van Nes, was a rotationplasty around the knee for femur deformities (2). This procedure enables the tibia to be fused to the femur, allowing for the removal of the pathological knee. However, there are several types of rotationplasty, each tailored to the specific needs of the patient. In 1986, Winkelmann expanded the classification of rotationplasties to include not only those for malignant tumors around the knee but also for those involving the hip (14).

Before 1986, when extensive tumors invaded the hip or pelvis, highly debilitating hindquarter amputations were often performed to save patients. Even today, hindquarter amputations are recommended for specific tumor types (15, 16). The literature highlighted the long-term poor quality of life of patients who underwent these amputations (15, 17, 18). Despite the undeniable significance of Winkelmann's rotationplasty, the literature on this topic remains surprisingly sparse, particularly regarding Type BII rotationplasties. Therefore, it is essential to fill this gap by conducting further studies that explore the intricacies of Winkelmann's rotationplasties. This article describes our experience with three patients who underwent a Winkelmann Type B2 hip rotationplasty in one case (14), and a modified surgery where the femur was fused to the sacroiliac joint in two cases.

## 2 Cases descriptions

This retrospective monocentric study was approved by the local ethics committee of the university hospital (2015/26JAN/025 Belgian registration number B403201523492). Written informed consent was obtained from the individuals and minor's legal guardian/next of kin for the publication of any potentially identifiable images or data included in this article.

### 2.1 Settings, time-frames and participants

The three patients were transferred to our institution in Brussels, Belgium. Patient #1 was first seen on September 18, 2022 and his follow-up lasted 6 months and 25 days. Patients #2 and #3 were taken care of on February 2, 2023 and March 31, 2023, respectively. All three patients underwent a Winkelmann Type B2 hip rotation with a modification of the femur fixation in two cases (14). The demographic characteristics of the patients are listed in Table 1.

**Table 1:** Patient demographics and surgical indications.

Patient	Age (years)	Sex	Surgical indication	Comorbidities	Localization	Medical history
#1	16	Male	Grade III conventional osteosarcoma	Active pneumonia (antibiotic therapy)	Left hip	Chemotherapy
#2	25	Male	Sepsis of THA mega-implant	Antibiotic therapy + spacer	Left hip	Left hip Ewing sarcoma
						Radiotherapy
#3	54	Male	High-grade liposarcoma recurrence	Smoker	Right hip	Resected grade III liposarcoma
						Radiotherapy

Antibiotic treatment of patient #2 consisted of Vancomycin (2 × 1.5 g/day) + Meronem (3 × 2 g/day). THA, total hip arthroplasty.

In 2021, patient #1 was diagnosed with Ewing sarcoma in another country. No radiotherapy was started, but chemotherapy was performed at the time, according to the Euro-ewing99 (19) protocol. The patient was subsequently transferred for the surgical procedure due to the unavailability of such specialized expertise in their country of origin. The most recent chemotherapy session occurred two weeks prior to the transfer. In the absence of a genetic EWSR1 rearrangement, a final diagnosis of Grade III conventional osteosarcoma was confirmed.

Patient #2 was referred from another country, where he had been treated for Ewing sarcoma 7 years earlier. A partial P2 resection and reconstruction with a hip prosthesis were performed, followed by post-operative radiotherapy. Although the patient remained disease-free, he unfortunately developed a chronic infection. Salvage surgery was attempted at our institution but was unsuccessful. As a result, a hip rotationplasty was proposed.

Patient #3 was treated initially in another institution for a “borderline benign lipomatous tumor” that was operated on and subsequently irradiated. He then suffered two recurrences that were treated in our hospital. He came back with yet another major recurrence that involved the thigh, the hip joint, and the iliac wing.

**Figure 1:**
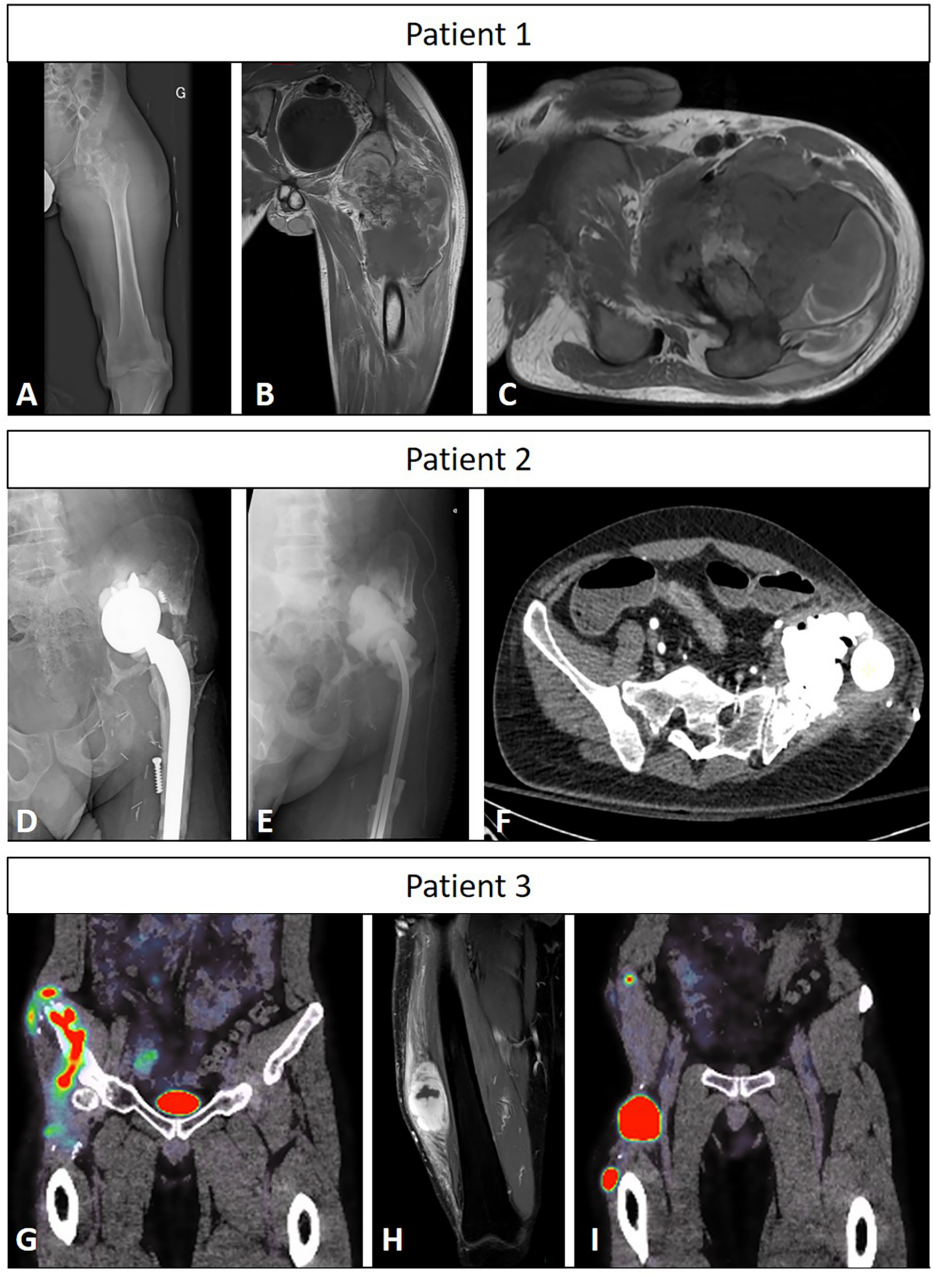
Preoperative imaging of the three patients. Patient #1: x-ray of the left hip showing a very destructive osteosarcoma **(A)**, frontal CT-scan of the same hip showing the extension and heterogeneity of the lesion **(B)**, and axial CT-scan around the hip of the same lesion **(C)** Patient #2: x-ray of the infected reconstruction of the left hip **(D)**, x-ray of the gentamycin spacer **(E)**, and transversal CT-scan showing the amount of cement and the spacer needed to fill the defect **(F)** Patient #3: Coronal CT-scan of the liposarcoma extension in the right hip and thigh **(G,I)** and soft-tissue magnetic resonance imaging acquisition showing another lesion on the lateral side of the thigh **(H)**.

The extension work-up for all three patients was negative at the time of referral. Figure 1 shows preoperative images of the patients.

### 2.2 Patient optimization and interventions

Patients were admitted to the orthopedic unit. They were given the opportunity to choose any psychological support they required. Patient #1 preoperative medical treatment was analgesic, anesthetic pre-medication and amoxicillin for his pneumonia. Patient #2 continued his supportive antibiotic and analgesic treatment until surgery. Patient #3 did not undergo any neoadjuvant treatment. He had received medical treatment such as radiotherapy prior to his transfer. None of the patients were taking chronic medication for any other medical condition.

Rotationplasty, although very uncommon, offers a superior quality of life (20–22), which is why it was chosen for all three patients. This type of rotationplasty was described by Winkelmann et al. in 1996 (14). Winkelmann B2 rotationplasty refers to a resection of the proximal femur combined with resection of the Enneking P2 and P3 pelvic areas (23).

All surgeries were conducted under general anesthesia, and all patients received supplementary continuous epidural anesthesia. All patients were positioned in a lateral decubitus position. Skin incisions were made as described by Winkelmann et al. All the surgical steps described below are illustrated in Supplementary Figure S1A.

The pathological soft tissues were resected along with the entire tumor, with particular attention given to preserving the superficial femoral artery and the sciatic nerve. After resection, the lower limb was rotated externally by 190°. For optimal ergonomic rehabilitation, 10° of external rotation and 5°–10° of abduction are required. In one patient, the common femoral artery and its bifurcation were resected with the tumor, and the superficial femoral artery was anastomosed to the external iliac artery. In Patient #1, the residual distal femoral diaphysis was shaped into a large step cut to match the bony surface of the lateral table of the iliac wing (P1 area). In Patients #2 and #3, following a P1–2 resection, the step cut was adjusted to fit a remnant of the sacroiliac joint. Four to five screws were sufficient to ensure stable compression and osteosynthesis between the rotated distal femur and the iliac wing.

The hamstrings were sutured to the abdominal wall muscles and the quadriceps to the proximal insertion of the gluteus medius and gluteus maximus muscles. To facilitate superficial sutures and avoid coverage defects, a de-epidermization method may be considered (24, 25). Skin closure consisted of Blair-Donati sutures. All three patients received an Incisional Negative Pressure Wound Therapy (INPWT) dressing (VAC Prevena Incision, KCI). This system improves healing, reduces complications, and facilitates dressing management (26–28).

The management of the superficial femoral artery must be decided intraoperatively, according to the morphology and fitting of the residual soft tissues. Shortening of the superficial femoral artery by resection and end-to-end anastomosis should be considered in cases where the artery is too long and risks being twisted or squeezed, or in cases where the artery is trapped inside the tumor (29).

Following surgery, all patients received standard intravenous antibiotic prophylaxis (Kefzol®), venous thromboembolism prophylaxis, and a standard analgesic regimen (level 1 and 2 analgesics as standard, with level 3 analgesics administered if needed). The antibiotic prophylaxis were administered as follow: 2 g at the anesthesia induction, 1 g every 3 h intraoperatively, 1 g at 8 h postoperatively and 1 g 16 h postoperatively. Each of the three patients spent a minimum of 24 h in the intensive care unit for immediate postoperative monitoring. They were then transferred to the orthopedic surgery department and subsequently to the rehabilitation department.

The surgeon in charge of these three clinical cases was an experienced orthopedic oncology surgeon at our institution. In all cases, the surgeon was always assisted by another orthopedic surgeon with experience in major reconstructions or oncological surgery. The senior assistant was always the same in all three cases, as was the team of vascular surgeons.

Postoperative rehabilitation adhered to a precise protocol designed to promote rapid mobilization and immediate neuromuscular re-education without compromising the integrity of the osteosynthesis. Daily wound care was meticulously performed, particularly given the proximity to the perineum. From a locomotor perspective, we advised caution during the initial weeks when sitting, as this position could impose significant mechanical stress on the osteosynthesis; without adequate bone consolidation, structural failure would have been likely. A perineal cushion was therefore utilized. Standing was strongly encouraged, always with the assistance of a physiotherapist or nurse, and with the aid of walking devices such as crutches or walkers, tailored to the physical capabilities of each patient. Psychologically, the opportunity for these three patients to undergo surgeries in close succession facilitated peer interaction, allowing them to better anticipate and comprehend the expected surgical outcomes. Once initial signs of bone consolidation were observed on x-rays, the process of fabricating an external prosthesis could commence. It is crucial to allow sufficient time for soft tissue remodeling around the reconstruction before proceeding with the fabrication of the external prosthesis.

### 2.3 Quality control

To minimize variability between cases, certain variables were strictly standardized across all patients. These consistent factors included the surgeon, operating assistance, surgical equipment, operating room, intensive care team, analgesic regimen, and nursing staff across the four departments involved (operating room, intensive care unit, orthopedic unit, and rehabilitation unit). Additionally, postoperative care, wound care, and the use of INPWT (incision negative pressure wound therapy) were meticulously adhered to, with ideal patient compliance. However, due to the individualized nature of this surgery, we also encountered significant differences, which are outlined below.

Patient #1 presented several notable differences e.g., tumor type, patient age, incorrectly administered neoadjuvant therapy, and concomitant pneumonia. Patient #2 also presented an infection of hip reconstruction mega prosthesis, no tumor, gentamycin spacer, antibiotic treatment, and an irradiated site. Lastly, patient #3 presented a tumor recurrence, previous surgery, a history of local radiotherapy and the superficial femoral artery trapped in the tumor.

### 2.4 Follow-up

Patients spent around 24 h in the intensive care unit when no complication occurred. This was followed by 15 days in the orthopedics department for wound control, pain management, and first physiotherapies. Finally, they were transferred to the rehabilitation department.

Orthopedic follow-up was relatively straightforward in the absence of complications. For the two patients with tumor pathology, close oncology follow-up was also maintained. A pelvic x-ray was performed upon discharge from intensive care to verify proper implant positioning. Additionally, a CT scan was conducted approximately 2–3 months postoperatively. In the event of complications, management was adapted according to the nature and progression of the issue.

Revalidation follow-up consisted of hydrotherapy, physiotherapy, occupational therapy, external appliances, nursing, and psychotherapy.

This case series has been reported according to the PROCESS Guidelines (30).

## 3 Results

### 3.1 Theoretical outcomes

Expected functional results were much better than after inter-abdominopelvic amputations (8, 31–35). The few references that do refer to this B2 Winkelmann procedure describe similar short-term results, but slightly poorer locomotor functional results, essentially due to the fact that hip abduction is impossible [compared with Type B3 rotationplasty (14, 20–22)] and hip extension is weaker due to the presence of the reversed knee. Good stability of the standing body is ensured by osteosynthesis as medial as possible (14, 21).

Winkelmann's B2 rotationplasty is designed to involve the resection of the P2 and P3 pelvic areas, along with the proximal femur. However, Winkelmann's classification should primarily serve as a surgical framework that can be tailored to each individual case. As a result, our case series sometimes involved modifications to the pelvic areas resected, such as resecting P1 while preserving P3. If the ischium can be spared, many mechanical and physiological functions, such as sitting and preserving the cavernous body of the penis, can be maintained.

### 3.2 Outcomes and follow-up

#### 3.2.1 Patient #1

The oncologic resection was classified as R0 by the pathologist. No vascular anastomosis was performed, and the artery was allowed to remain in a natural loop within the pelvis. This patient experienced a significant number of postoperative complications (details provided below). As a modification to the initial management plan, we were able to preserve the P3 pelvic zone. Additionally, a Spica cast was applied for pain relief.

**Figure 2:**
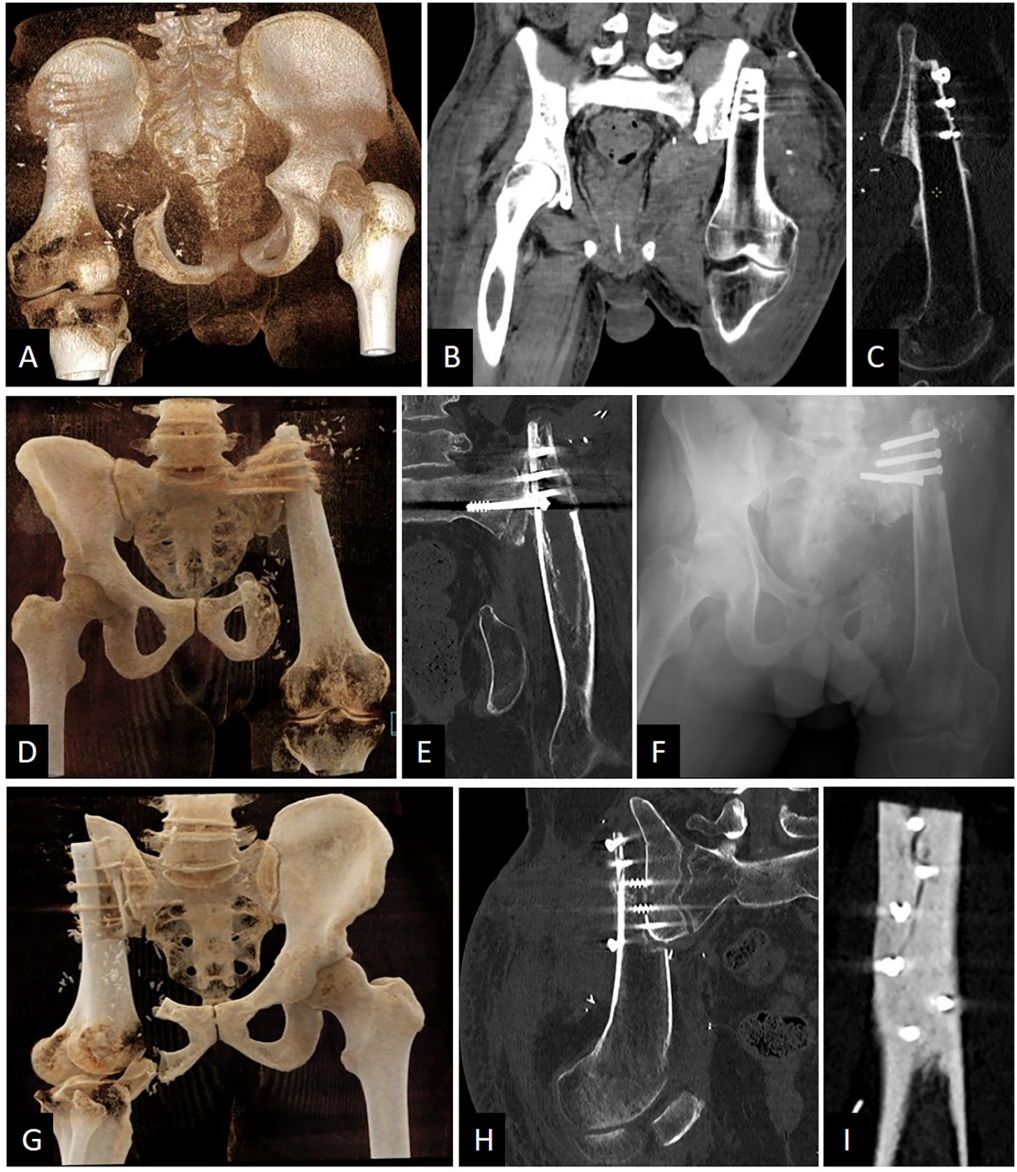
Relevant postoperative imaging of the three patients. **(A)** Patient #1. Posterior CT-scan 3D reconstruction showing that P3 was preserved. **(B)** Patient #1. CT-scan coronal view of the pelvis. Stabilization was ensured by five screws in P1. **(C)** Patient #1. CT-scan sagittal view of the osteosynthesis showing good consolidation ongoing after 4 months post-surgery. **(D)** Patient #2. Anterior CT-scan 3D reconstruction showing that P3 was preserved and P1 was resected. **(E)** Patient #2. CT-scan coronal view of the pelvis showing screws going in the sacral wing. **(F)** Patient #2. Anteroposterior x-ray view of the pelvis. Four screws were sufficient for stabilization. **(G)** Patient #3. Anterior CT-scan 3D reconstruction showing that P3 was partially preserved and P1 was largely resected. **(H)** Patient #3. CT-scan coronal view of the pelvis showing screws going in the sacral wing. **(I)** Patient #3. CT-scan sagittal view of the external femoral cortex fixed to the residual P1 and sacral wing. A small visible crack changed the rehabilitation postoperative care due to a risk of major fracture.

After 4 months postoperatively, the patient was painless. He was able to walk with his external prosthesis for 1 km with the help of one crutch at the end of his walk. No limping or Trendelenburg gait when walking was reported. He was able to climb at least 30 stairs with two crutches. No discomfort was described regarding his external prosthesis. He complained about residual pain when he tried to stand up. Imaging showed an excellent ongoing consolidation between the femur's diaphysis and the iliac wing (Figures 2A–C). The musculoskeletal tumor society score (MSTS) was calculated at 23. Scar was well closed, sciatic nerve functions were fully preserved and the hip flexion ranged from 0° to 80° in active range of motion (ROM) and 90° in passive ROM. Supplementary Video S1 of the gait and walking pattern of patient #1 three months postoperatively.

Unfortunately, this patient suffered of multiple pulmonary metastasis disease 7 months postoperatively.

#### 3.2.2 Patient #2

In this case, as well as in the subsequent one, resection of P1 was necessary. Consequently, the femur was fixed to the lateral aspect of the sacroiliac joint. Compared to the preoperative plan, we successfully preserved P3 during the pelvic resection. Due to the extent of the pathology, P1 had to be removed, and no vascular anastomosis was performed. To date, the patient has not experienced any postoperative complications. Imaging results indicate good early consolidation and no signs of screw loosening (Figures 2D–F).

At 3.5 months of follow-up, his musculoskeletal tumor society score (MSTS) was calculated at 22. The patient developed cholecystitis, which was treated by laparoscopic cholecystectomy. The patient walked with two crutches, the scar was well closed, sciatic nerve functions were fully preserved and his hip ROM was ranged from 0° to 50° passively and actively.

#### 3.2.3 Patient #3

Oncologic resection was classified as R0. This patient needed a resection and end-to-end anastomosis of the artery because a segment of the superficial femoral artery was trapped in the tumor. De-epidermization was also performed during closure.

P3 was partially preserved and, because of the pathology volume, P1 had to be removed. After one month postoperatively, a small crack in the femur was observed on the CT-scan (Figure 2G–I). This changed the rehabilitation planning. No revision surgery was performed, but a delayed full weight-bearing was decided.

Three months postoperatively, the patient was ambulating with two crutches and was permitted to bear 10 kg of weight on the operated limb. The scars were well-healed, and sciatic nerve function was fully preserved, although the patient still experienced some discomfort. Hip range of motion (ROM) ranged from 0° to 50°, both passively and actively. However, in this case, the femur was fixed with excessive external rotation. The Musculoskeletal Tumor Society score (MSTS) was calculated to be 18.

At the end of their follow-up, every patient obtained bone consolidation.

### 3.3 Intervention adherence and complication

In terms of postoperative recommendations, every patient demonstrated exemplary compliance.

### 3.4 Complications and adverse events

In accordance with the Clavien-Dindo Classification (36), the postoperative complications of patients are listed below (Table 2).

**Table 2:** Complications summaries for every patient.

Complication	Clavien-dindo grade	Post-op day	Management	Medical unit
Patient 1				
Pain	1	3	Level 3 analgesics	ICU
Decompensated cardiorespiratory failure	1	0–17	IV crystalloids	ICU
Ionic disorders	1	8–18	Infusion for ionic correction	ICU
Sacral bedsore	3B	8	VAC change	OR
Urinary tract infection	2	10	Antibiotics IV	ICU
Scar infection	3B	12	Lavage & scar revision	OR
Opioid tolerance	1	13	Dose increase	Pediatric unit
Polymicrobial sepsis	2	10–105	Multi-antibiotics IV	Pediatric unit
Wound dehiscence	3B	21	Scar revision	OR
Left iliac pseudoaneurysm & collection	3B	26	Embolization & drainage	Interventional radiography
Stenosis of the femoral artery	3B	27	Surgical release of compression	OR
Pulmonary metastasis	3B	129	Wedge resection	OR
Anemia	2	130	Transfusion	ICU
Hemothorax and hypotension	3A & 2 & 1	131	Drain & transfusion & IV saline	ICU
Sepsis	2	135	Antibiotics IV	Pediatric unit
Pulmonary metastases	5	179	Palliative care	Pediatric unit
Patient 2				
				
Patient 3				
Neuropathic pain	1	19	Opioids intake	Rehabilitation unit
Wound necrosis	3B	28	Scar resection	OR
Femur light crack	1	33	Delayed total weight bearing	Rehabilitation unit

The type of complication, the Clavien-dindo Grade of the latter, the time of occurrence, the management and the concerned medical unit are listed in this table. ICU, intensive care unit; OR, operating room; IV, intravenous; VAC, vacuum assisted closure.

## 4 Discussion

This article describes a small case series recently treated in our institution. This very extensive surgery allows to avoid hindquarter amputations. Type B2 rotationplasty requires superior surgical skills and a good knowledge of pelvic anatomy to be performed successfully. However, with careful patient selection, it remains a very interesting option that may offer patients a good quality of life, as long as a strong multidisciplinary team is involved.

Inter-abdominopelvic disarticulation is a heavy surgical procedure, leading to many complications and an extremely reduced quality of life (15, 17, 18, 37, 38). Despite an esthetic image that can be shocking for the patient, rotationplasty offers a much better quality of life (39). Indeed, there is no real amputation stump, healing is easier, and the long-term tolerance is much better. In addition, preserving the ankle motor control allows more precise and physiological control of the ROM of the “neo-knee” gait (21, 22, 40). Patients have little or no Tredelenburg gait, and therefore no vicious compensation. By medializing the limb, B2 rotationplasty brings the operated limb closer to the patient's center of gravity, improving balance control and Pauwels’ balance.

Rotationplasty is a technique that is closely aligned with the principles of limb salvage surgery. Initially described by Van Ness and his team for treating pathologies around the knee, this procedure was primarily developed for pediatric oncological cases (2, 6, 41). The cerebral plasticity of younger patients allows for better adaptation to the neuromotor changes associated with this type of surgery. The favorable functional outcomes reported have encouraged some teams to broaden the indications for rotationplasty (42), expanding its application to different anatomical regions (43) and, in certain cases, extending it to particular populations (35). Rotationplasty around the knee is well-documented in the literature, with long-term functional outcomes being particularly favorable, especially in pediatric populations (44, 45). Application of this technique on other areas than the knee is possible and can also lead to great outcomes. This paper gives a new insight on Winkelmann B2 rotationplasties and their modifications.

Due to the resected tumor lesions, we had to adapt our surgical strategy (for patients #2 and #3) and modify the Winkelmann Type B2 rotationplasty. Specifically, we decided to fix the femur directly on the sacrum wing/sacroiliac joint instead of the P1 zone of the iliac bone. This modification affects the Pauwels balance because it alters the lever arm between the pivot represented by the neo hip joint and the moment of force exerted by body weight. The medialization of this pivot in comparison with a fixation on the P1 zone reduces its importance. Consequently, the resultant forces applied to the neo hip joint will be weaker than in the case of P1 fixation, requiring the muscles to generate less force, thereby reducing the risk of Trendelenburg gait (14). Furthermore, reducing leverage and Trendelenburg gait might also improve bone consolidation at the osteosynthesis site. This medialization may result in a conflict between the femoral condyles and the ischium if the P3 zone is preserved. Unfortunately, there are no studies in the literature on gait analysis for the Winkelmann Type B2, unlike the Type B3 (22) and Van Ness (11, 40). These two reconstructions (patients #2 and #3) prompt us to reconsider Winkelmann's classification by proposing a variant of the B2 rotationplasty. Specifically, although B2 rotationplasty is typically described as resecting the P2 and P3 areas with fixation on P1, we introduced modifications in these cases. For these patients, we resected P1, P2, and a portion of P3, altering both the resection and fixation sites. This adapted approach could be termed the *modified Winkelmann B2* or *Winkelmann B2 with sacral fixation*.

In Winkelmann rotationplasty, the hip joint is replaced by the knee joint, which only allows movements in a single plane (flexion/extension). As a result, patients have no abduction or adduction and often have trouble maintaining a sitting position. However, despite these limitations, the Winkelmann rotationplasty still yields satisfactory functional results (20, 22). Given that these surgeries are limb salvage procedures of last resort, often employed for extensive pathologies, we believe it would be challenging and potentially hazardous to establish geometric standards applicable for all patients. These interventions must be evaluated on a case-by-case basis. Nevertheless, we can consistently aim for the anatomical objective of positioning the neo-hip at the same level as the contralateral native hip. This alignment ensures optimal physiological tension in the residual soft tissues sutured at the end of the operation. Additionally, medialization of the neo-hip may enhance biomechanics and reduce muscular fatigue associated with Pauwels balance, particularly as this will involve muscle groups other than the gluteals in our case.

Regarding the method of osteosynthesis, we prioritize bone biology. Instead of extensively preparing (and potentially de-periostealizing or de-vascularizing) the entire distal femur to accommodate a large lateral plate, we prefer to utilize a limited number of screws to stabilize the construct.

This study is not without limitations as it was carried out on a small cohort of patients with a short follow-up. It is important to keep in mind that the MSTS score is supposed to increase as revalidation progresses. This is usually a long process for this type of pathology. Nevertheless, our calculations are currently within an acceptable range compared with Winkelmann's previous articles studying his Type BI rotationplasty. Indeed, the MSTS scores of his rotationplasties were between 21.6 and 24.4 (21), and ours were rated at 23, 22, and 18 from the longest to the shortest follow-up, respectively.

The surgical indication, preoperative planning, postoperative follow-up, and assessments were all discussed and determined in a multidisciplinary setting involving radiologists, oncologists, anatomopathologists, infectiologists, orthopedists, and vascular surgeons. Additionally, all surgeries were performed by the same team of surgeons, and the follow-up and rehabilitation were conducted by a consistent team of intensivists, rehabilitation physicians, physiotherapists, and nurses. This ensured a shared experience among the entire multidisciplinary team.

A prerequisite for all types of rotationplasties is the preservation of the intact sciatic nerve (39). In the 1986 description of his procedure, Winkelmann (14) recommended resection of part of the vascular bundle and end-to-end anastomosis, as he felt that the vascular loop arrangement was more prone to complications. The literature on vascular management is scarce. In 2008, Mahoney et al. (29) found no significant difference in terms of complications between the two methods (resection-anastomosis vs. loops), but their studies focused only the Type A1 rotationplasty. In 2013 Tiwari (46) published a report on three patients who underwent resection and anastomosis of the vascular bundle and showed good results. Our experience with Type B2 rotationplasty has led us to make case-by-case decisions based on residual soft tissues and femoral artery tension at the time of osteosynthesis. Of note, when the artery is sufficiently loose in the operating field without being able to twist or loop, no vascular anastomosis should be considered at this stage.

In order to create more consistent guidelines, it is necessary to have access to larger series or to undertake literature reviews. Unfortunately, the literature on this subject is currently too sparse.

## 5 Conclusions

The Winkelmann Type B2 rotationplasty demands a multidisciplinary team with expertise in managing complex cases, as it presents significant technical challenges and must be customized for each patient. The functional outcomes and quality of life in our patients surpass those of individuals who underwent hip disarticulations. Additionally, fixation of the femur to the sacrum is achievable while maintaining good functionality, comparable to a standard B2 procedure.

## Data Availability

The original contributions presented in the study are included in the article/Supplementary Material, further inquiries can be directed to the corresponding author.

## Supplementary material

Supplementary Figure S1 Step-by-step Surgical Intervention. **(A)** Surgical landmarks. The patient is positioned in lateral decubitus. The white asterisk indicates the anterior superior iliac spine in all images. **(B)** Tumor dissection and isolation of critical structures. The yellow arrow points to the femoral nerve, and the blue arrow indicates the femoral vessels. Unfortunately, the femoral nerve had to be sacrificed as it was infiltrating the tumor. **(C)** Specimen removal. The surgical specimen is carefully extracted following the diaphyseal femoral cut, with the yellow arrow highlighting the sciatic nerve. **(D)** Post-tumor removal. The blue arrow shows the preserved femoral vessels (nerves are not visible in this image). **(E)** Evaluation of preserved structures and surgical field. Examination of the cut planes and hemostasis. The yellow arrow marks the sciatic nerve, and the blue arrow indicates the femoral vessels. **(F)** Femoral preparation. A specific step-cut of the proximal femur is performed to optimize the geometrical fit of the femur against the iliac wall (black circle). The yellow arrow highlights the sciatic nerve, and the blue arrow shows the femoral vessels. **(G)** Pathologist’s macroscopic view of the proximal femur specimen. **(H)** Histological Haematoxylin/Eosin staining, confirming the tumor type as osteosarcoma. **(I)** Reconstruction step. Limb rotation is performed, assessing vascular structure tension and slackness, and testing the femur-to-ilium fit. A 10° external foot rotation is essential. **(J)** Immediate postoperative image. The left heel is positioned at the contralateral knee level, confirming the 10° external foot rotation. **(K)** Three months postoperatively, demonstrating good active limb function.

Supplementary Video S1 Limb function three months postoperatively.
