# Supplementary figures and images for: Case Report: Winkelmann hip rotationplasty as a last-resort solution
Source: Front Surg. 2025 Jan 7;11:1433291. doi: 10.3389/fsurg.2024.1433291 (PMC11756526; doi:10.3389/fsurg.2024.1433291)

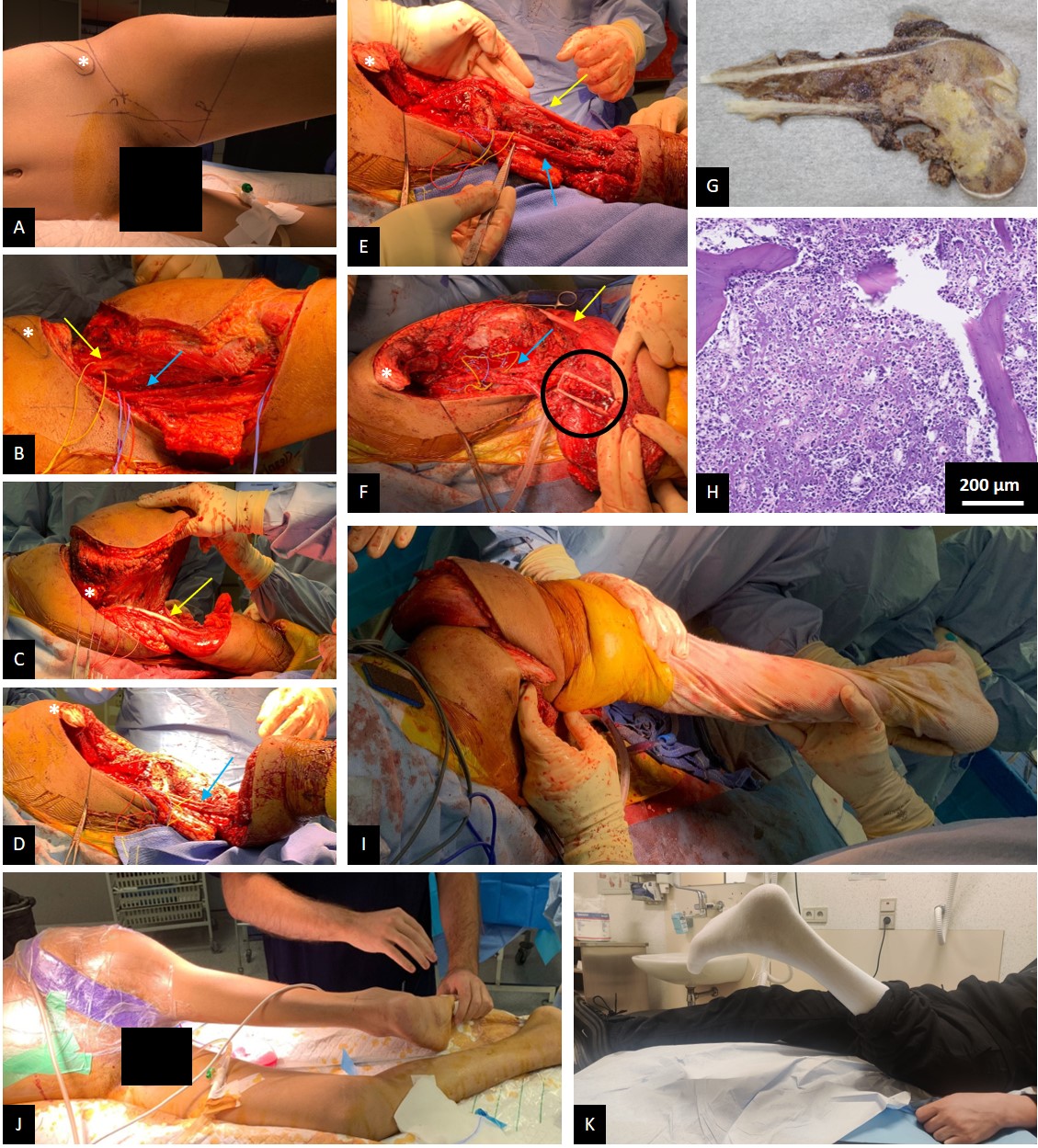

Supplement: Supplementary Figure S1 — Step-by-step Surgical Intervention. (A) Surgical landmarks. The patient is positioned in lateral decubitus. The white asterisk indicates the anterior superior iliac spine in all images. (B) Tumor dissection and isolation of critical structures. The yellow arrow points to the femoral nerve, and the blue arrow indicates the femoral vessels. Unfortunately, the femoral nerve had to be sacrificed as it was infiltrating the tumor. (C) Specimen removal. The surgical specimen is carefully extracted following the diaphyseal femoral cut, with the yellow arrow highlighting the sciatic nerve. (D) Post-tumor removal. The blue arrow shows the preserved femoral vessels (nerves are not visible in this image). (E) Evaluation of preserved structures and surgical field. Examination of the cut planes and hemostasis. The yellow arrow marks the sciatic nerve, and the blue arrow indicates the femoral vessels. (F) Femoral preparation. A specific step-cut of the proximal femur is performed to optimize the geometrical fit of the femur against the iliac wall (black circle). The yellow arrow highlights the sciatic nerve, and the blue arrow shows the femoral vessels. (G) Pathologist’s macroscopic view of the proximal femur specimen. (H) Histological Haematoxylin/Eosin staining, confirming the tumor type as osteosarcoma. (I) Reconstruction step. Limb rotation is performed, assessing vascular structure tension and slackness, and testing the femur-to-ilium fit. A 10° external foot rotation is essential. (J) Immediate postoperative image. The left heel is positioned at the contralateral knee level, confirming the 10° external foot rotation. (K) Three months postoperatively, demonstrating good active limb function. [file Image1.jpeg]
